# Surgical Outcomes of Posterior Arch Resection of the Atlas Alone for Upper Cervical Myelopathy in Patients With Athetoid Cerebral Palsy: A Report of Two Cases

**DOI:** 10.7759/cureus.73946

**Published:** 2024-11-18

**Authors:** Yuji Endo, Kazuyuki Watanabe, Koji Otani, Yoshihiro Matsumoto

**Affiliations:** 1 Department of Orthopedic Surgery, Fukushima Medical University School of Medicine, Fukushima, JPN; 2 Department of Research for Spine and Spinal Surgery, Fukushima Medical University School of Medicine, Fukushima, JPN

**Keywords:** athetoid cerebral palsy, atlanto-dental interval, case report, posterior arch resection, upper cervical myelopathy

## Abstract

This study reports two cases of upper cervical myelopathy with athetoid cerebral palsy (ACP) treated with posterior arch resection. Both patients exhibited good long-term outcomes with no recurrence at five or 10 years. Decompression surgery can be effective for ACP patients with upper cervical spine lesions.

## Introduction

Cervical myelopathy in patients with athetoid cerebral palsy (ACP) presents specific challenges related to abnormal movement and muscle tone and requires a different treatment strategy to that of general cervical myelopathy. Treatment planning requires a comprehensive approach that takes into account the patient's general condition and motor function [1,2]. ACP develops at a young age and requires long-term management due to its rapid progression. On the other hand, generalized cervical myelopathy is more common in middle-aged and older people and progresses slowly. Although conservative treatment is ineffective in improving myelopathy in patients with ACP, surgical treatment is often recommended. Due to the characteristics of the disease, such as involuntary movements, decompression alone may predispose patients to kyphotic deformities or the recurrence of stenosis of the cervical spine, so fusion surgery is frequently needed [2]. Despite its merits, spinal fusion is associated with a potential reduction in the range of motion, thereby potentially compromising the activities of daily living (ADL). In addition, fusion may cause breathing and swallowing difficulties and implant failure resulting from involuntary movements [3-6].

In this report, we present two cases of ACP associated with upper cervical myelopathy at the C1 level that were effectively managed with posterior arch resection of the atlas alone, with long-term follow-up.

## Case presentation

Case 1

A 57-year-old woman with ACP complained of muscle weakness in her left upper and lower extremities and gait disturbance for six years. The patient's symptoms gradually worsened, and surgery was scheduled. The intensity of the athetotic movements according to the Mihara classification was grade 3 [7], and the preoperative Japanese Orthopedic Association (JOA) score [8] was six.

**Figure 1:**
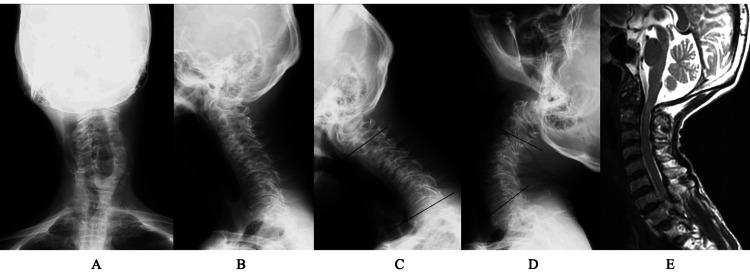
Preoperative image findings of case 1 A plain X-ray of the cervical spine in a neutral position (A, anterior-posterior view; B, lateral view) showed scoliotic change with a decrease in lordosis. Lateral view with flexion (C) and extension (D) showed changes in lordotic angle from -11° to 35°. The sagittal view of MRI T2WI (E) showed spinal cord compression at the C1 level. There was no considerable stenosis at the middle to lower levels T2WI: T2-weighted image

Preoperative radiographs revealed an atlanto-dental interval (ADI) of 2.4 mm in the neutral position and degenerative changes in the middle and lower cervical spines. The lateral view with flexion and extension showed a lordotic angle defined as a C2-7 angle from -11° to 35°, and no evidence of axial subluxation or instability in flexion and extension exists. MRI at the neutral position indicated stenosis at the C1 level (Figure 1). The axial image showed spinal cord compression and central stenosis at the C1 level from the posterior only.

We aimed to preserve the range of motion of the cervical spine, considering that spinal cord compression is primarily caused by the posterior arch of the atlas. Therefore, we decided that the resection of only the posterior arch would be sufficient to achieve decompression.

**Figure 2:**
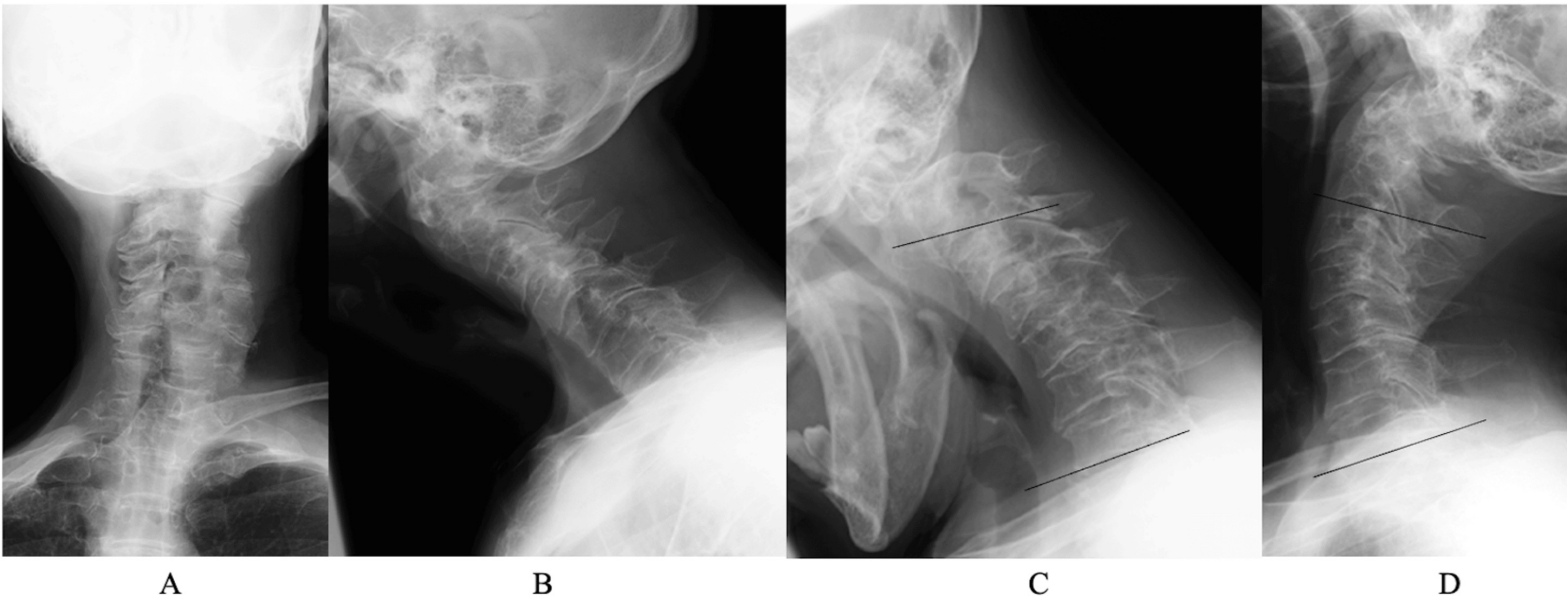
Five-year postoperative image findings of case 1 A plain X-ray of the cervical spine in a neutral position (A, anterior posterior view; B, lateral view) showed an increase in spondylotic changes compared to the preoperative findings. Lateral view with flexion (C) and extension (D) showed changes in lordotic angle from -3° to 18° and no evidence of atlantoaxial subluxation

The patient was unable to walk before surgery but could walk with a walker in the early postoperative period, and her ambulatory function was maintained for five years postoperatively. The motor weakness of the upper limb was slightly improved and maintained. However, radiographs taken five years postoperatively showed an increase in the lordotic angle, -3° to 18°, and worsening degenerative changes (Figure 2).

Five years postoperatively, the patient had a JOA score of 10 points, a 36% improvement rate, and maintained her walking ability with the use of a walker.

Case 2

A 62-year-old woman with ACP presented with a four-year history of gait disturbance and was diagnosed with cervical myelopathy. Her walking difficulties progressed owing to spasticity, and surgery was planned. According to the Mihara classification, the intensity of athetotic movements was grade 3, and the preoperative JOA score was 10.5.

**Figure 3:**
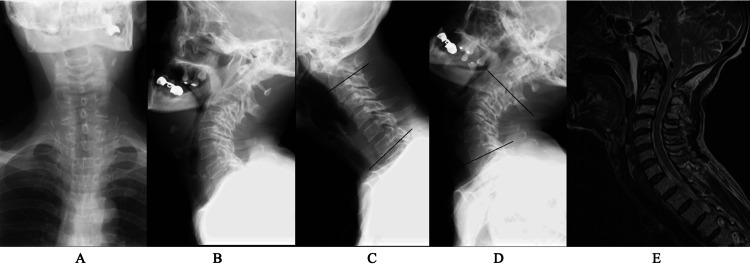
Preoperative image findings of case 2 A plain X-ray of the cervical spine in a neutral position (A, anterior posterior view; B, lateral view) showed less spondylotic changes. Lateral view with flexion (C) and extension (D) showed changes in lordotic angle from 5° to 60°, and atlanto-dental interval (ADI) is 5.4 mm at flexion and 2.6 mm at extension. The sagittal view of MRI T2WI (E) showed spinal cord compression at the C1 level. There was no considerable stenosis at the middle to lower levels T2WI: T2-weighted image

Preoperative radiography revealed an ADI of 2.8 mm in the neutral position. The ADI during flexion was 5.4 mm, and the extension was 2.6 mm. Hence, we assessed the instability. The lordotic angle ranged from 5° during flexion to 60° during extension. MRI indicates moderate stenosis at the C1 level. There was prominent posterior compression with intramedullary signal changes and central stenosis and no stenosis in the middle or lower cervical spine (Figure 3).

For case 2, we used the same approach of resecting only the posterior arch for the same reasons as in case 1, aiming to preserve the range of motion of the cervical spine, given that spinal cord compression was again primarily due to the posterior arch.

**Figure 4:**
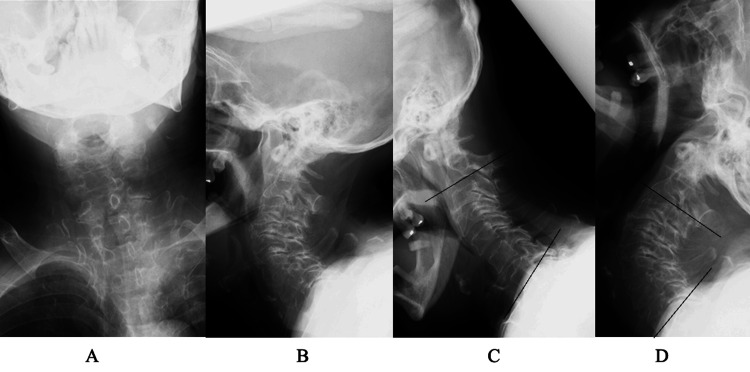
Five-year postoperative image findings of case 2 A plain X-ray of the cervical spine at a neutral position (A, anterior posterior view; B, lateral view) showed an increase in spondylotic changes compared to preoperative findings. Lateral view with flexion (C) and extension (D) showed changes in lordotic angle from 25° to 65°

One year after the surgery, the patient could walk with a walker and assistance, and her JOA score was 13 points, with a 38% improvement rate. However, after 10 years, her JOA score decreased to 11 because her upper limb movement and sensation worsened. Her improvement rate was 8%; however, she was still able to walk using a walker. Radiographs did not show kyphosis progression; the C2-7 angle was 25° at flexion and 65° at extension, even though degenerative changes progressed. The ADI showed almost no change at 10 years postoperatively (Figure 4).

## Discussion

Since the first report of cervical myelopathy associated with ACP in 1962, numerous accounts of surgical strategies, mainly for stenosis at the middle to lower levels, have been published [9]. In contrast, the frequency of stenosis in the upper cervical spine is relatively low, and many of these cases have been treated with fusion of the atlantoaxial spine alone or extensive fusion involving the occiput to the lower cervical spine [10]. Therefore, there are few reports on the results of patients treated with C1 posterior arch resection alone; in addition, long-term follow-ups have not been reported [11].

In both cases reported here, the mobility of the atlantoaxial joint was maintained by avoiding fusion surgery, and the long-term results were favorable without the progression of severe kyphotic deformity.

In patients with ACP, stenosis of the upper cervical spine often occurs 10 years after that of the middle and lower cervical spines [12]. Repetitive involuntary movements of the head and neck result in the degeneration of the middle and lower cervical spines and spondylotic changes, which in turn decrease the range of motion of the middle and lower cervical spines, thereby increasing the mechanical stress on the upper cervical spine.

In contrast, when the instability of the atlantoaxial joint occurs because of the slack of the transverse ligament, mechanical stress on the atlantoaxial joint is likely to be repeated because of involuntary head and neck movements, leading to stenosis at the same site.

We evaluated two distinct cases of cervical spine pathologies. The first patient exhibited no instability of the atlantoaxial joint; however, kyphotic changes were observed. This kyphotic deformity is compensated by the hyperextension of the upper cervical spine during forward gaze, which induces the degeneration of the upper cervical spine. Conversely, the second case presented no kyphotic changes in the cervical spine but only had instability at the atlantoaxial joint. Cases of atlantoaxial joint subluxation without kyphosis or limited range of motion have been reported; we believe that these cases do not progress with posterior arch resection. In patients with ACP and cervical myelopathy at the C1 level, posterior arch resection may be a sufficient therapeutic intervention, even in cases of kyphotic deformity or atlantoaxial joint instability.

Compared to decompression alone, fusion surgery has been shown to have good clinical results; however, there is a risk of implant failure, and long-term results have reported the recurrence of symptoms at adjacent segments in a number of patients [11]. However, in some cases, atlantoaxial instability, which was not evident preoperatively on imaging, became apparent after C1 posterior arch resection, leading to the worsening of myelopathic symptoms [13]. Therefore, appropriate surgical options should be developed based on the patient's condition. However, it is not clear in which cases the long-term outcome of decompression alone is better, and it is important to accumulate cases.

In summary, we report the long-term results of two patients with upper cervical myelopathy and ACP treated with posterior arch resection of the atlas alone. They achieved good improvement in symptoms after surgery, which was maintained for 5-10 years. However, there have been reports of symptom recurrence after more than 10 years of follow-up [11]. Further careful follow-ups are required.

## Conclusions

Even in patients with ACP who are prone to chronic stress in the cervical spine due to involuntary movements, decompression surgery alone may lead to good long-term outcomes in patients with lesions involving only the upper cervical spine. However, there are few reports, and further accumulation is needed.

## References

[1] Duruflé A, Pétrilli S, Le Guiet JL, Brassier G, Nicolas B, Le Tallec H, Gallien P (2005). Cervical spondylotic myelopathy in athetoid cerebral palsy patients: about five cases. Joint Bone Spine.

[2] Singh K, Samartzis D, Somera AL, An HS (2008). Cervical kyphosis and thoracic lordoscoliosis in a patient with cerebral palsy. Orthopedics.

[3] Kim KN, Ahn PG, Ryu MJ, Shin DA, Yi S, Yoon DH, Ha Y (2014). Long-term surgical outcomes of cervical myelopathy with athetoid cerebral palsy. Eur Spine J.

[4] Wong AS, Massicotte EM, Fehlings MG (2005). Surgical treatment of cervical myeloradiculopathy associated with movement disorders: indications, technique, and clinical outcome. J Spinal Disord Tech.

[5] Watanabe K, Otani K, Nikaido T (2017). Surgical outcomes of cervical myelopathy in patients with athetoid cerebral palsy: a 5-year follow-up. Asian Spine J.

[6] Jameson R, Rech C, Garreau de Loubresse C (2010). Cervical myelopathy in athetoid and dystonic cerebral palsy: retrospective study and literature review. Eur Spine J.

[7] Mihara H, Kondo S, Kohno M, Niimura T, Onari K, Hachiya M (2008). [Clinical analysis of reoperation following surgical treatments for cervical spondylotic myelopathy accompanied with athetoid cerebral palsy] (Article in Japanese). Rynsho Seikeigeka.

[8] Japanese Orthopedic Association (1994). Scoring system for cervical myelopathy. J Jpn Orthop Assoc.

[9] Anderson WW, Wise BL, Itabashi HH, Jones M (1962). Cervical spondylosis in patients with athetosis. Neurology.

[10] Pollak L, Schiffer J, Klein C, Mirovsky Y, Copeliovich L, Rabey JM (1998). Neurosurgical intervention for cervical disk disease in dystonic cerebral palsy. Mov Disord.

[11] Haro H, Komori H, Okawa A, Shinomiya K (2002). Surgical treatment of cervical spondylotic myelopathy associated with athetoid cerebral palsy. J Orthop Sci.

[12] Mihara H, Tatara Y, Niimura T, Sekiya T, Goda A (2019). [Surgical management for cervical disorder in cerebral palsy patients: countermeasures and learn from experience] (Article in Japanese). Seikei Saigaigeka.

[13] Goda A, Miraha H, Tatara Y (2020). [Surgical outcomes for upper cervical lesions in patients with athetoid cerebral palsy] (Article in Japanese). J Spine Res.

